# Opportunities and Challenges in Using Electronic Health Record Systems to Study Postacute Sequelae of SARS-CoV-2 Infection: Insights From the NIH RECOVER Initiative

**DOI:** 10.2196/59217

**Published:** 2025-03-05

**Authors:** Hannah L Mandel, Shruti N Shah, L Charles Bailey, Thomas Carton, Yu Chen, Shari Esquenazi-Karonika, Melissa Haendel, Mady Hornig, Rainu Kaushal, Carlos R Oliveira, Alice A Perlowski, Emily Pfaff, Suchitra Rao, Hanieh Razzaghi, Elle Seibert, Gelise L Thomas, Mark G Weiner, Lorna E Thorpe, Jasmin Divers, RECOVER EHR Cohort

**Affiliations:** 1 Department of Population Health New York University Grossman School of Medicine New York, NY United States; 2 Applied Clinical Research Center The Children's Hospital of Philadelphia Philadelphia, PA United States; 3 Louisiana Public Health Institute New Orleans, LA United States; 4 Department of Genetics The University of North Carolina at Chapel Hill School of Medicine Chapel Hill, NC United States; 5 Department of Epidemiology Columbia University Mailman School of Public Health New York, NY United States; 6 Department of Population Health Sciences Weill Cornell Medicine New York, NY United States; 7 Division of Infectious Diseases Department of Pediatrics Yale University School of Medicine New Haven, CT United States; 8 Division of Health Informatics Department of Biostatistics Yale University School of Public Health New Haven, CT United States; 9 Blooming Magnolia Los Angeles, CA United States; 10 Department of Medicine The University of North Carolina at Chapel Hill School of Medicine Chapel Hill, NC United States; 11 Department of Pediatrics University of Colorado School of Medicine and Children's Hospital Colorado Aurora, CO United States; 12 Department of Neuroscience USC Dornsife College of Letters, Arts and Sciences Los Angeles, CA United States; 13 Clinical and Translational Science Collaborative of Northern Ohio Case Western Reserve University Cleveland, OH United States; 14 Department of Foundations of Medicine New York University Long Island School of Medicine Mineola, NY United States; 15 See Acknowledgments

**Keywords:** COVID-19, SARS-CoV-2, Long COVID, post-acute COVID-19 syndrome, electronic health records, machine learning, public health surveillance, post-infection syndrome, medical informatics, electronic medical record, electronic health record network, electronic health record data, clinical research network, clinical data research network, common data model, digital health, infection, respiratory, infectious, epidemiological, pandemic

## Abstract

The benefits and challenges of electronic health records (EHRs) as data sources for clinical and epidemiologic research have been well described. However, several factors are important to consider when using EHR data to study novel, emerging, and multifaceted conditions such as postacute sequelae of SARS-CoV-2 infection or long COVID. In this article, we present opportunities and challenges of using EHR data to improve our understanding of long COVID, based on lessons learned from the National Institutes of Health (NIH)–funded RECOVER (REsearching COVID to Enhance Recovery) Initiative, and suggest steps to maximize the usefulness of EHR data when performing long COVID research.

## Introduction

Postacute sequelae of SARS-CoV-2 infection, colloquially known as long COVID, refers to ongoing, relapsing, new symptoms, or other health effects after the acute phase of SARS-CoV-2 infection (ie, present 4 or more weeks after the acute infection). Because long COVID is heterogeneous in presentation and may occur after a mild or even asymptomatic infection, it has posed unique and significant challenges for clinical research, including an overarching lack of data that are accessible and analyzable in a rigorous and reproducible manner to inform diagnostic criteria and care guidelines.

SARS-CoV-2 continues to impact populations globally with new and recurrent infections. While studies have provided a wide range of estimates regarding the proportion of patients with COVID-19 who develop long COVID [1], even conservative estimates point toward an enormous burden. Without established treatments targeting underlying pathophysiologic mechanisms, patients and clinicians have largely focused on treating symptoms [2] and managing organ damage, highlighting an urgent need to expand our knowledge of COVID-19 infection’s long-term effects.

Electronic health records (EHRs) contain large quantities of clinical data that can be used for research without significant delay, making them an important data source for accelerated insight into emerging health conditions. However, challenges associated with leveraging EHR data for research can become amplified when mobilizing rapidly to study these conditions, particularly when they are multifaceted and lack clear hallmark traits or biomarkers.

The National Institutes of Health (NIH)–funded REsearching COVID to Enhance Recovery (RECOVER) Initiative was launched in 2021 [3] to advance research into long COVID. Broadly, RECOVER supports prospective clinical studies of adult and pediatric cohorts and real-world data studies that leverage EHR networks to study long COVID. These studies have been developing and validating algorithms to identify patients with long COVID within EHR networks for clinical and epidemiological characterization, risk factor prediction, and identification of prevention and treatment opportunities. RECOVER’s real-world data efforts are led by 3 participating EHR research networks: the National COVID Cohort Collaborative (N3C); the National Patient-Centered Clinical Research Network (PCORnet); and PEDSnet, a pediatric learning health system within PCORnet. These networks are coordinated by a Clinical Science Core (CSC) at NYU Langone Health.

In this paper, we highlight opportunities and challenges of using EHR data to improve our understanding of long COVID, with a focus on analytical resources and scenarios we’ve encountered as US-based researchers, and conclude with some next steps for maximizing the usefulness of EHR data for long COVID research. Similar considerations may pertain to other chronic infection–associated diseases, that is, complex multisystemic conditions often occurring in the context of infection with other pathogens, including myalgic encephalomyelitis/chronic fatigue syndrome (ME/CFS).

## EHR-Based Research Networks

### Overview

The widespread adoption of heterogeneous health information systems across the United States hinders large-scale EHR-based research efforts. To overcome this, federal funding from the Patient-Centered Outcomes Research Institute (PCORI), Centers for Disease Control and Prevention (CDC), and NIH has facilitated the growth of networks that transform data into a common data model to facilitate aggregation across participating institutions. These networks are often referred to as clinical research networks (CRNs).

While such networks are a focus of this paper, we recognize that other successful international [4-7] and national models have been leveraged for large-scale long COVID studies. Domestically, the Department of Veterans Affairs (VA), which operates the largest integrated health care system in the United States, has made important contributions characterizing long COVID, its burden, and risk after reinfection, immunization or nirmatrelvir (Paxlovid) use [8-11]. The interoperability across many VA hospitals and clinics allows for data aggregation without the extensive harmonization or loss of data depth that usually accompanies a research network, though VA patients (military experience, mostly men) are not representative of the general population. Similarly, researchers have used claims data and outpatient laboratory results from UnitedHealth, a large health insurance provider, to characterize risk of long COVID in commercially insured patients [12]. The addition of claims data enables better understanding of health care use, inequities in access to care and costs associated with long COVID, though quantifying the larger economic burdens and disparities requires incorporation of data sources outside the health care system.

While researchers with access to EHR data from large clinical networks or health systems may bypass some challenges below, many national and international EHR-based collaborations wrestle with similar obstacles.

### Opportunities

CRNs provide several benefits for conducting clinical research. Most significantly, they unify patient data that may otherwise be distributed across multiple health care facilities and facilitate access to large sets of timely, longitudinal patient information. This enables broad hypothesis generation, signal identification, and feasibility analysis, which is highly advantageous for emerging conditions like long COVID as such analyses can be conducted at a scale, pace, and cost that surpass traditional research methods [13]. Because CRNs use data generated during health care delivery, they are also well-positioned to study how practices evolve in ways that directly impact patients. For example, EHR data can be used to evaluate uptake and patterns of use for new diagnostic codes introduced to capture long COVID [14], ME/CFS, and postural orthostatic tachycardia syndrome (POTS).

To overcome the lack of semantic interoperability across EHR systems and facilitate analysis, most CRNs map source data to common data schemas, such as the Observational Medical Outcomes Partnership (OMOP) [15] or PCORnet common data models [16], which provide frameworks for data standardization and are optimized for large-scale longitudinal data analysis. The popularity and extensibility of common data models helped CRNs to quickly adapt them for novel disease research at the beginning of the pandemic [17]. Use of a common data model also enables distributed network queries and analyses behind institutional firewalls, as well as centralization of the data for advanced artificial intelligence and machine learning [18].

In a relatively short time, a large amount of scientific literature has been generated regarding the clinical characterization and epidemiology of long COVID, yet efforts to synthesize this body of research have been hampered by the inconsistency of methodologic approaches, study definitions, and findings. Research conducted using a shared infrastructure or data model promotes reproducible research and exchange of clinical and variable definitions that can be harmonized and tested across multiple health systems. For example, a recent collaboration between RECOVER and NIH’s All of Us study demonstrated success in reusing long COVID definitions across OMOP data environments, enabling reproduction of N3C’s definition within the All of Us database [19].

Along the same lines, CRNs are often well-positioned to foster multidisciplinary collaboration [20]. They have streamlined contracting and collaboration processes that expedite research while reducing regulatory burden, and can also provide an efficient vehicle for patient recruitment into prospective cohort studies and clinical trials.

### Challenges

Some broad challenges come with using CRNs for population health research and may directly impact research into long COVID, where data quality, depth, and timeliness are crucial. These largely arise from the need to standardize data across multiple institutions, which may result in a loss of detail and context from the originating EHR [21]. For example, while the OMOP and PCORnet data models support documentation of patient cause of death and discharge disposition, not all do; furthermore, not all sites collect and report these data. Analyses can also be limited by not having information such as provider specialty, medication indication, or drug dosing consistently abstracted from the EHR. However, as described earlier, the extensibility of many common data models means that new concepts can be incorporated to close priority gaps.

Aggregating data within a CRN can mask important heterogeneity due to differences in how health systems and individual practitioners provide and document care. One example is the variation in how visit data are represented across individual health care facilities. The definition of an “encounter” in a given health system’s data has more to do with business rules than clinical care; for example, some systems create a separate encounter record for each day of an inpatient stay, while others use a single record to capture the entire hospitalization. As most variables in EHR data are captured at the visit level, inconsistency in the definition of encounters can lead to errors when analyzing clinical observations. Thus, to accurately classify types of visits and to quantify visits across sites, careful standardization is required [22]. Ongoing governance, documentation, and input from data experts at contributing institutions are essential to bridge syntactic and semantic differences [21].

Timeliness of data delivery represents an additional challenge. While data from CRNs are generally rapidly available, there is still a latency from participating institutions. Data must be mapped, transformed, transmitted, quality checked, and potentially resubmitted with corrections, which takes time and collaborative effort. Delays may also result from the need to incorporate data standards for new concepts (such as the long COVID *ICD-10* [*International Statistical Classification of Diseases, Tenth Revision*] code U09.9, which was not introduced until late 2021).

Finally, real-world data present specific limitations compared with traditional research data. These include data missingness, which has been described extensively [23] and can exist for various reasons (eg, patient discontinuation of care or data points not being systematically captured in EHRs). EHR data are also subject to biases like selection bias [24], where patients accessing care may be sicker, more likely to be insured, and able to take time off work and afford transportation [13,25]. Data from nonacademic institutions, and other settings that may lack robust data systems such as nursing homes, are often underrepresented in CRNs. Careful study designs, informatics-informed approaches to transforming raw clinical data into analytical datasets [26], techniques to supplement structured EHR data with items extracted from free text notes such as social determinants [27], and advanced statistical approaches are often required to minimize bias.

## Identifying Patients With Long COVID Within EHRs

### Overview

Robust case definitions are essential to disease surveillance, enabling consistent use of data from collection through analysis and across population subgroups, time, and geographic jurisdictions. However, Long COVID still lacks a uniform case definition, and multiple frameworks have emerged with varying timeframes and symptoms [25]. This is partly due to the heterogeneity of long COVID’s clinical presentation, which includes substantial differences between adults and children [28] and hundreds of potential signs and symptoms. The National Academies of Sciences, Engineering, and Medicine’s recent report A Long COVID Definition provides updated recommendations around diagnosis, noting that long COVID can impact any organ system; follow acute infection of any severity, including asymptomatic infection; be continuous from acute infection or delayed in onset; and present as incident or worsening, mild or severe, and persistent or intermittent [29]. These broad guidelines leave room for ambiguity, and as there is no diagnostic test for long COVID, their application at the point of care involves clinical judgement, experience, and thorough differential diagnosis to establish probable causality.

Finally, we are still learning about long COVID’s disease course and trajectory, how it relates to development of clinical conditions such as diabetes, kidney disease, and POTS, and how its characteristics may evolve over time. While these complexities underlie all long COVID research, EHR-based research is particularly well-positioned to inform and validate potential definitions.

### Opportunities

Researchers working with EHR data operationalize case definitions by creating computable phenotypes, which algorithmically identify cohorts of interest within an EHR system [30]. Generating, adapting, and validating computable phenotypes can be complex, even for well-characterized diseases and conditions, so multiple approaches for identifying patients with long COVID have evolved both nationally and internationally. These include building computable phenotypes using “rules-based” approaches or prespecified logic-based queries to search for specific criteria such as diagnosis codes, medications, or laboratory results based on clinical literature and expertise [31-33], and data-driven or inductive approaches, including machine learning (ML) to automatically classify long COVID without detailed prespecification of the inclusion or exclusion criteria [34,35]. These approaches can also be combined, leveraging insights from ML to shape rules-based definitions.

The lack of a formal case definition, limited SARS-CoV-2 testing information, and large amounts of rich longitudinal data within EHRs make ML approaches particularly attractive. Supervised ML models can be trained on sets of patients that are certain or highly likely to have long COVID, such as patients assigned a long COVID diagnostic code, and then used to identify patients with probable long COVID who may not have a documented or readily recognized diagnosis [35]. ML and other statistical approaches have also been applied to recognize incident diagnoses that appear more frequently in cohorts with a recent history of COVID-19 infection than those without [36,37].

Although the EHR does not capture every case (or every recurrence) of acute SARS-CoV-2 among its patients, there is still significant opportunity to use EHRs to study the potential effect of SARS-CoV-2 reinfection on risk of long COVID among those patients with multiple recorded cases. Hadley et al [38] found that across SARS-CoV-2 variants, the incidence of new long COVID diagnoses after reinfection is lower than after the initial SARS-CoV-2 infection. Bowe et al [11] found that though risk is lower with more recent variants, it is cumulative across infections. As multiple reinfections become more common, there will be continual opportunities to reevaluate these findings in the context of new variants and subvariants.

Both rules-based and ML techniques are being used to identify potential clusters or subphenotypes of long COVID [39-41]. For example, an analysis by Zhang et al [41] identified four distinct subphenotypes (including cardiac and renal, musculoskeletal, and nervous system) and specific risk predictors for each. Clustering analyses may uncover diverse symptoms that have common biological causes, and in establishing distinct groupings around them, facilitate diagnosis and treatment targeted toward specific subtypes [42].

### Challenges

Within the EHR, Long COVID may not be well-documented due to diagnostic complexities, as described earlier. The initial focus among clinicians on respiratory symptoms and widespread lack of knowledge around what were initially considered to be “atypical” presentations, such as postexertional malaise, ME/CFS, POTS, and dysautonomia, also contribute to missingness of relevant diagnoses within the EHR [43], which is compounded by the absence of or delay in introduction of *ICD-10* codes for many of these conditions, including the U09.9 code for long COVID itself**.** Such manifestations can significantly impact daily function but may not be considered attributable to previous COVID-19 infection (or misdiagnosed as mental health issues), leading to their underrepresentation in research [43].

Health care professionals may be subject to implicit biases that impact documentation of patient information (eg, social determinants and symptoms) within the EHR [24]. This may also lead to underestimation of long COVID for certain populations, such as racial or ethnic minorities. Indeed, U09.9 diagnostic codes have been found to be more common among patients that are non-Hispanic White and living in areas of low poverty compared with the overall COVID-19–positive population, potentially reflecting differences in access to care and other interactions with the health care system [14]. Although we found value in using visits to a long COVID clinic as an early proxy for a long COVID diagnosis code [35], both of these markers have biases and should not be considered a gold standard for long COVID identification [44]. There is also substantial need to complement patient-reported symptoms with clinically characterized ones [45] so that physicians are not “gatekeepers” of documented symptoms.

Given that long COVID is likely underdiagnosed and underdocumented, computable phenotype algorithms must supplement formal U09.9 diagnoses with other patient data following acute SARS-CoV-2 infection. However, identifying acute infections is also not straightforward. Diagnosis codes such as the ICD-10 code U07.1 have demonstrated variable sensitivity and positive predictive value [46,47], which may be exacerbated by “rule out” diagnoses or improper coding. Even when diagnoses are correct, the true timing of the initial infection with respect to the date of the diagnosis code may be uncertain [44], and we found this to complicate EHR-based calculations of long COVID incidence. Limited availability of SARS-CoV-2 testing during the early phase of the pandemic, widespread testing at nonclinical settings (eg, kiosks and pharmacies), and the later popularity of at-home tests have all contributed to low confidence in accurately capturing SARS-CoV-2 infection (and reinfection) history within the EHR, and amplified potential misclassification of long COVID patients without documented acute COVID-19 infection. We note, however, that misclassification of patients as uninfected is an issue that extends beyond EHR-based studies [48], broadly impacting long COVID research.

ML methods are also subject to obstacles, including the lack of a validated test set for long COVID and dependency of results on the training set used, which may limit reproducibility (although, as demonstrated by the collaboration between RECOVER and All of Us, ML algorithms can be replicated in new environments) [19]. ML models must be interpretable, so predictions can be explained instead of only existing within a “black box.”

## Determining Risk Factors for Long COVID Using EHRs

Risk factors for long COVID include demographic characteristics, lifestyle behaviors, underlying comorbidities, and social and environmental factors. Identifying risk factors can guide screening and prevention efforts, particularly in highly susceptible groups, and potentially facilitate tailored long COVID treatments.

### Opportunities

EHR-based studies are primed to quickly reveal possible associations and risk factors for conditions like long COVID due to large volumes of available data and ability to stratify by various subgroups. For example, Rao et al [28] leveraged PEDSnet’s geographical and clinical breadth to identify risk factors such as age, severity of acute COVID-19 infection, and comorbid conditions. Potential biological variables that are well-defined in EHRs and therefore, more reliably available for application in adjusted analyses include age, sex, BMI, and blood pressure, and, indeed, these variables have been shown to confound the evaluation of causal pathways for long COVID [49-51].

Where data of interest are missing from the EHR, linkage to external sources can increase data quality and completeness. Statewide vaccination or vital record data can be acquired and linked at the patient or area level to enhance the accuracy of vaccine status and COVID-19 outcomes [52,53]. Linkage with geographic information systems (GIS) can address questions about complex networks of long COVID burden that pertain to certain geographic regions, including the so-called “exposome” [54] even down to ZIP codes and neighborhoods [13,14]. Patient-reported outcomes from patient portals and surveys can improve the completeness of information on demographic, social, and behavioral factors such as race, ethnicity, substance use, exercise frequency, and smoking status [13,55]. To enhance completeness of data such as outpatient medication use, linkage to claims data can be an effective strategy. Data of interest may also be captured within free-text clinical notes, images, and other scanned documents, requiring application of natural language processing tools, including cutting-edge language models [27], or manual chart review to incorporate them.

Within EHR-based studies, several analytic approaches can address confounding and effect modification and help measure mediating influences. Researchers can match comparison groups using various approaches, including propensity scores for known confounders. In addition, causal mediation analysis methods may more accurately explain observed associations between risk factors and long COVID development.

Designing studies with appropriate comparison groups for analyses of risk factors can be challenging. If comparison groups are matched too closely, relevant differences in concurrent risk factors and outcomes may be masked. If groups are not matched well enough, then the impact of confounders may overwhelm the ability to detect differences associated with the characteristic of interest. Researchers using real-world data can conduct sensitivity analyses under different assumptions of risks and outcomes. For example, Hill et al [56] leveraged 3 different control groups and 2 definitions of long COVID to enhance an investigation of risk factors within N3C. This agility is especially useful with a novel condition such as long COVID, where our understanding is rapidly evolving. Comparison groups can be iteratively modified, and analyses rerun as new information or criteria emerge.

EHR data, especially within the context of a CRN, also facilitates the study of risk factors within rare populations and subgroups (eg, peoples indigenous to the United States). Such groups often experience a disproportionate burden of COVID-19 complications due to socioeconomic and structural factors [51,57], but are generally not well-represented in prospective studies and randomized controlled trials [58]. EHR data can similarly help overcome the methodological constraints and recruitment challenges of rare disease research [13], providing an opportunity to study rare conditions and outcomes and facilitating clinical trial cohort selection.

Finally, EHRs provide rich datasets for pragmatic clinical trials and target trial emulation to identify potential therapies [59,60]. For example, there is inconclusive data on the efficacy of Paxlovid for preventing or treating long COVID [61]; novel approaches such as target trial emulation using EHR data can help evaluate its efficacy and determine which patients should be prioritized for treatment based on risk factors for severe long COVID subphenotypes. The ability to quickly evaluate and personalize potential treatments is highly advantageous given the large burden of long COVID and its heterogeneous presentation. Because target trial emulation better reflects real-world medication adherence (or application of other interventions), comparing the results of emulated and clinical trials, and examining how the methods and populations differ, may yield insights into generalizability of clinical trial results and sources of bias within observational analyses.

### Challenges

Limitations outlined earlier, such as data missingness, selection bias, and implicit biases, can directly impact the ability to evaluate risk factors using EHR-based analyses. Potential risk factors can be rigorously evaluated if well-defined and well-captured, but many important risk factors may not be completely, meaningfully, or consistently recorded within the EHR.

For example, race and ethnicity are important demographic risk factors for long COVID [51], but these data can be missing in over 25% of patient records [55,62]. Lack of robust, structured data on social and behavioral determinants of health, such as physical activity levels, smoking status, and alcohol use [13,51,62] may necessitate supplementation with survey results or data extracted from clinical notes as described above. EHR-based assessments of symptom severity and improvement may also be of limited use without these approaches.

Imperfect data capture also impacts our ability to establish proper comparison groups. This notably includes the shrinking population of patients without previous SARS-CoV-2 infection [63]. In addition to being challenging to identify, control patients identified by negative lab tests may actually be in worse health than those with mild COVID-19 infection, likely due to routine testing of patients within the emergency department or inpatient settings. Another example applies to our work examining obstructive sleep apnea (OSA) as a risk factor for long COVID [64]. A comparison group of patients ostensibly without OSA may include some with undiagnosed, subclinical, or undocumented OSA, and sizable numbers of misclassified patients would lead to underestimation of effect size on long COVID. Similarly, slight improvements in recognition of ME/CFS throughout the pandemic may have resulted in some individuals with prepandemic long COVID–like symptoms (eg, unrefreshing sleep and postexertional malaise) having their illness misclassified as SARS-CoV-2–related. Appropriate comparison groups must be carefully considered.

The pandemic disrupted typical health care use and documentation patterns. Clinic closures and reduced access to specialty care may have masked diagnoses of chronic conditions and comorbidities and led some patients with long COVID, particularly those with milder presentations, not to seek care. This biases EHR data, especially from the first year of the pandemic, toward underascertainment of health issues; prepandemic controls (who we can say with certainty have not had COVID-19 infection) may have higher rates of clinical events than pandemic-era patients. Similarly, research about the effectiveness of vaccinations in reducing the risk of long COVID is hampered by widespread distribution in nonclinical settings [65] and reinforces the importance of ongoing data exchange between EHRs and vaccination registries in addition to dedicated linkage efforts.

These challenges also apply to potential confounding, which is particularly important within observational research on risk factors and causal mechanistic pathways for long COVID. Certain potential confounders, such as age or underlying health conditions, are easier to adjust for than others. The severity of acute COVID-19 infection, which appears in some studies to be directly associated with the risk of developing long COVID [49,50,66], is a particularly illustrative example. More severe COVID-19 infection, whether it results in hospitalization or not, may result in increased frequency of diagnosis and monitoring of long COVID and higher data density for affected patients within the EHR. These biases may contribute to the underrepresentation of asymptomatic, mild, or moderate acute COVID-19 infections, as well as greater clinician awareness of long COVID in the context of severe illness (although most cases occur among nonhospitalized patients) [43]. Analyses stratified by acute illness severity are important to differentiate between nonhospitalized and hospitalized patients.

## Discussion

As SARS-CoV-2 and our susceptibility to infection evolve, sustained research efforts are needed to build our knowledge of long COVID and evaluate its presentation over time. Within a short timeframe, the use of EHR networks to study long COVID has grown rapidly, nationally and internationally. The immediate need to advance our understanding of long COVID must be balanced with a thoughtful methodological approach that leverages the many strengths of EHR data while recognizing their limitations. EHRs have been used to characterize long COVID and its subphenotypes [14,32,40,41,45], estimate its burden [28,67], identify risk factors [49-51,66], and evaluate SARS-CoV-2 vaccine efficacy for long COVID prevention [8,53,68]. Although we anticipate that the breadth and rigor of this research will increase as computable phenotypes improve through iterative validation and data linkage becomes more extensive, several innovations to effectively wield EHR data, such as improved accuracy and consistency of recording of long COVID-specific diagnoses, application of ML to identify patients with long COVID and harmonized data analyses across CRNs, have already begun increasing the capacity of investigators to study long COVID and, importantly, augment research on long COVID subphenotypes.

EHRs can be instrumental in supplementing randomized controlled trials to evaluate treatments and preventative strategies such as vaccinations, but novel approaches such as target trial emulation have not yet widely extended to long COVID. Increased data linkage to vaccination registries, survey data, and other sources can further enrich EHR-based analyses, providing more complete patient outcomes and medical history data. As recruitment into prospective RECOVER studies increases, efforts are underway to link EHR data with participant study data, enhancing both datasets for validation and analysis.

We envision a near future where the mutual benefits of clinical and real-world data research collaborations have been optimized for synergistic learning. EHRs can identify study participants and generate hypotheses that can be prospectively tested. They can also inform clinical trial feasibility, generate preliminary data around specific populations, therapeutics, and outcomes of interest, and inform the interpretation of clinical trial results through trial emulation. However, strengthening this interchange will require new workflows, collaboration, and time.

We caution that despite the dedication of the research community and high expectations for EHRs to quickly advance our understanding of long COVID, sustained, collaborative efforts are needed for this work. New treatments and vaccines are still being introduced, requiring process modifications for extraction and standardization of data. Complex clinical traits require careful development of computable phenotypes and rigorous data quality evaluation to identify specific misclassification risks. Continued coordination is necessary to converge upon reliable, repeatable insights and yield effective strategies for long COVID.

## Abbreviations

CDC: Centers for Disease Control and Prevention
CSC: clinical science core
CRN: clinical research network
EHR: electronic health record
GIS: Geographic Information Systems
ICD-10: International Statistical Classification of Diseases, Tenth Revision
ME/CFS: myalgic encephalomyelitis/chronic fatigue syndrome
ML: machine learning
N3C: National COVID Cohort Collaborative
NIH: National Institutes of Health
OMOP: Observational Medical Outcomes Partnership
OSA: obstructive sleep apnea
PCORI: Patient-Centered Outcomes Research Institute
PCORNet: National Patient-Centered Clinical Research Network
POTS: postural orthostatic tachycardia syndrome
RECOVER: REsearching COVID to Enhance Recovery
VA: Department of Veterans Affairs
